# Safety and Quality Improvement of NaCl-Reduced Banana and Apple Fermented with *Lacticaseibacillus paracasei*

**DOI:** 10.3390/foods14010051

**Published:** 2024-12-27

**Authors:** Jose M. Martín-Miguélez, Josué Delgado, Irene Martín, Alberto González-Mohino, Lary Souza Olegario

**Affiliations:** 1 Higiene y Seguridad Alimentaria, Facultad de Veterinaria, Instituto Universitario de Investigación de Carne y Productos Cárnicos (IProCar), Universidad de Extremadura, 10003 Cáceres, Spain; jmmm@unex.es (J.M.M.-M.); iremartint@unex.es (I.M.); 2 Tecnología de los Alimentos, Facultad de Veterinaria, Instituto de Investigación de Carne y Productos Cárnicos (IProCar), Universidad de Extremadura, 10003 Cáceres, Spain; albertogj@unex.es (A.G.-M.); laryolegario@unex.es (L.S.O.)

**Keywords:** lactic acid bacteria, lacto-fermentation, fruit, CATA, RATA, *Escherichia coli* O157:H7

## Abstract

Food preservation techniques changed during the industrial revolution, as safer techniques were developed and democratized. However, one of the simplest techniques, adding salt, is still employed in a wide variety of products, not only as a flavor enhancer but as a method to allow for the controlled fermentation of products such as fruits. The objective of the present study consists of evaluating the quality of different salt-reduced fermented fruits through the application of the lactic acid bacteria (LAB) *Lacticaseibacillus paracasei* and vacuum, as well as assessing the LAB as a preventive measure against *Escherichia coli* O157:H7. To achieve this goal, microbial plate count techniques, the evaluation of the physicochemical characteristics, and Check-All-That-Apply/Rate-All-That-Apply sensory analyses were performed on bananas and apples individually fermented at 30 °C for 2 and 7 days, respectively. Additionally, a challenge test using *E. coli* as pathogenic bacteria was performed. The characteristics of each fruit determined the efficiency of the LAB’s protective activity. LAB-inoculated batches controlled the growth of *E. coli* in both salted fruits, but this pathogenic bacterium in the apple case was controlled even in the salt-reduced batch. Sensorially, only inoculated fermented apples found a reduction in off-flavor and old fruit smell; however, higher acceptability was found in the salt-reduced with LAB batches of both fruits. Thus, *Lacticaseibacillus paracasei* proved to be a cheap, easy, and feasible protective method that can ensure a protective strategy on salt-reduced fermented apples and should be studied particularly for different fruits.

## 1. Introduction

Food preservation techniques that were employed by our ancestors have become popular in the later years of modern cooking; recipe books were the main knowledge dissemination tool used in professional and amateur kitchens [1]. However, wild fermentation presents a diverse number of hazards that need to be considered to increase the safety of foodstuffs [2,3]. The general knowledge of these books lies in fermented fruits or vegetables with 2% salt and temperatures close to 30 °C [4]. Salt has always been one of the main ingredients able to preserve foods, though, nowadays, it is related to hypertension and cardiovascular diseases [5]. Specifically, the European Food Safety Authority (EFSA) has recommended limiting the sodium intake to 2.0 g per day, due to its involvement in cardiovascular diseases [6]. This salt limitation intake is a problem all around the world, and any action conducted to reduce salt is necessary due to the elevated levels of salt ingested through ultra-processed foods [7,8,9,10].

Salt is a key ingredient in meat and dairy products, such as dry-cured sausages or ripened cheeses, in which its content has been studied to be reduced, evaluating its safety and quality changes [11,12,13]. Fruits and vegetables have usually been conserved either dried or mixed with salt and other ingredients [14]. Nowadays, fermented fruits and vegetables are becoming a trend due to the nutritional benefits that lactic acid bacteria (LAB) can develop, in addition to an improvement in their sensory characteristics and safety [15,16]. Traditional fruit-based fermented beverages consist of the wild fermentation of the raw material, usually developing an alcoholic drink, or through the fermentation with complex microbiota associations such as kefir or kombucha [16,17]. Non-alcoholic actual fermented drinks are pasteurized and inoculated with LAB to direct the fermentation [18]. However, whole fermented fruits and vegetables are still elaborated with a percentage of salt above 2% to guarantee the stability of the product, as this allows for the development of LAB above other spoiling microorganisms, in addition to being a preserver itself [19,20,21]. Microorganisms and salt substitution have been employed in different vegetable industries to reduce the sodium content [22]. However, there is a lack of research on salt-reduced fermented fruits with the introduction of preservative cooking technologies widely employed in industries and professional kitchens, such as vacuum or the use of microorganisms present in the Qualified Presumption of Safety (QPS) list of the EFSA [4,23,24]. Fermentations with microorganisms are currently being carried out in professional kitchens all over the world, so the importance of this study is to shed some light on their hazards and possible solutions [25]. Special emphasis is placed on how the use of different food matrices or variations in fermentation conditions can affect the quality and food safety of the final product.

The objective of the present study consists of introducing a microbial control technique such as vacuum packaging to two different salt-reduced fermented fruits, banana and apple. Also, the addition of LAB is evaluated as a microbial control strategy and a quality enhancer. This study corresponds to an interesting investigation for industries and professionals which aim to obtain healthier fermented fruits, with reduced sodium, high quality, and safety control.

## 2. Materials and Methods

### 2.1. Fruit Samples

Cavendish bananas and Golden apples were obtained from local stores to evaluate the functionality of the employed technology. The fruits were peeled and cut into 1 cm^3^ cubes using clean, disinfected materials (ethanol 70% *v*/*v*), further being fermented under vacuum conditions.

### 2.2. Fermentation

Extensive preliminary research involving 162 trials was conducted to determine key parameters for the lactic acid fermentation process. These trials focused on identifying the most suitable LAB species for inoculation, establishing the lowest salt concentration necessary to initiate fermentation, and determining the shortest fermentation duration required to produce a perceptible acid taste. A trained panel of 4 food science researchers was employed to select the ideal elaboration method. The acidity and alcohol production in fermented banana and apple were influenced by varying salt concentrations ranging from 0% to 2%, tested at 0.25% increments. Banana fermentation exhibited higher acidity at lower salt percentages, while apple fermentation showed increased acidity and reduced alcohol production at higher salt percentages. The fruits were vacuum packed and fermented in 90 μm thick pouches (with oxygen transmission rate under 70 cc per square meter per day at 23 °C and 0% RH, and water vapor transmission rate under 10 g per square meter per day at 38 °C and 90% RH). Developed vacuum fermentation was conducted at 30.0 ± 1.0 °C, with specific conditions for each fruit: bananas were fermented for 3 days with 0% salt, while apples were fermented for 7 days with 1% salt. Control bananas and apples were fermented at 2% salt.

### 2.3. Bacterial Strains

This study utilized cheese-isolated *Lacticaseibacillus paracasei* (LAB 335) from the Food Hygiene and Safety research group at the University of Extremadura. This strain was selected to enhance product quality under various salt concentrations and fermentation durations. *L. paracasei* was cultivated in Man, Rogosa, and Sharpe broth (MRS) from frozen stock (−80 °C) preserved in the same media with 20% (*w*/*v*) glycerol. After a two-day incubation at 30.0 ± 0.1 °C, the culture broth was centrifuged, and an initial concentration of 6 log CFU/g was calculated and verified on MRS agar.

### 2.4. Physicochemical Analysis

The pH and water activity (a_w_) were measured with the aid of a digital pH meter (Hach Lange Spain S.L.U., Barcelona, Spain) and an a_w_-meter (Novasina AG, Lachen, Switzerland), respectively [26]. Total soluble solids using a refractometer were expressed in °Brix [27]. Only the *E. coli* non-inoculated samples were analyzed in triplicate and with repetition, similar to the microbiological analysis.

### 2.5. Microbial Analyses

**Figure 1:**
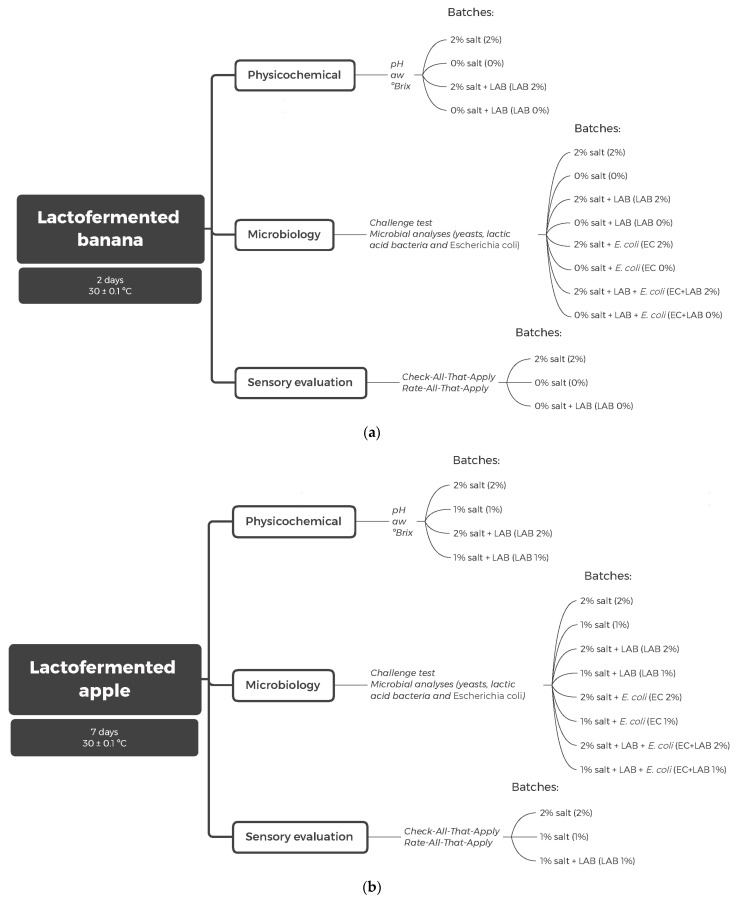
Scheme of the experimental design performed for banana (**a**) and apple (**b**).

A challenge test with *Escherichia coli* O157:H7 (CECT 7619) was performed, which belongs to the Spanish Type Culture Collections (Madrid, Spain) and was originally isolated from fruits. To prepare the inoculum, *E. coli* was grown in Brain Heart Infusion (BHI; Conda Pronadisa, Madrid, Spain) at 37 °C for 24 h. Then, the culture broth was centrifuged in 50 mL polypropylene tubes (Scharlau Chemie S.A., Barcelona, Spain) at 10,621 g for 5 min. An initial concentration close to 2 log CFU/g was initially calculated and checked on Violet Red Bile Glucose Agar (VRBG) before inoculation. Eight distinct batches were prepared for each fruit, comprising four batches with *E. coli* and four with *L. paracasei*, varying in salt concentration and bacterial presence. The batches included combinations of salt levels with or without *E. coli* and *L. paracasei*, allowing for a comprehensive analysis of the effects of salt concentration and bacterial inoculation on the fermentation process (Figure 1a,b).

Microbiological analyses were conducted by taking 10 g from every batch with 90 mL of 1% (*w*/*v*) peptone water (Conda Pronadisa). Decimal dilutions of peptone water were then prepared, and agar plates from various media were inoculated corresponding to the sampled microbial group. MRS agar was utilized for sampling LAB, and plates were incubated at 30 °C for 48 h under microaerophilic conditions, facilitated by enclosing the plates in a plastic bag to create an oxygen-deprived environment. Malt Extract Agar (MEA; composed of 20 g malt extract (Conda Pronadisa), 20 g glucose (Labbox Labware S.L., Barcelona, Spain), 1 g bacteriological peptone (Conda Pronadisa), 20 g bacteriological agar (Conda Pronadisa), and 1 L distilled water) was employed to assess the yeast load, with an incubation period of 48 h at 25 °C. Tryptone Bile X-Glucuronide Agar (TBX; Conda Pronadisa) was incubated at 37 °C for 24 h to determine the presence of *E. coli*. Triplicates were performed per batch, and the experiment was repeated twice within two consecutive months.

### 2.6. Sensory Analyses

The sensory characteristics of the LAB-uninoculated batches and the LAB-inoculated salt-reduced batches of both fruits, as detailed in Figure 1a,b, were evaluated using the Check-All-That-Apply (CATA) and Rate-All-That-Apply (RATA) techniques. For this, sixty-eight consumers between students and personal staff from 18 to 64 years of age were recruited at the Hospitality School of Salamanca (Salamanca, Spain). They were asked to select all the characteristics they perceived in the evaluated sample and indicate the intensity using a 5-point scale anchored at 1 = slightly to 5 = extremely. Samples were presented randomly, and water was offered to clean the palate between samples.

The CATA/RATA questionnaire used consisted of a list of 23 attributes that were generated using descriptive sensory terms from previous studies on fermented fruits and three preliminary sessions with a focus group of 4 researchers in food science from the University of Extremadura [28,29]. Additionally, consumers indicated how much they liked the samples through a seven-point structured hedonic scale, with scores ranging from 1 (dislike extremely) to 7 (like extremely).

This study was conducted following the 1964 Helsinki Declaration, and the protocol was approved by the Delegation of the Bioethics and Biosafety Commission of the University of Extremadura (Delegación de Comisión de Bioética y Bioseguridad de la Universidad de Extremadura) with the approval number 71/2016.

### 2.7. Data Analyses

The data were first analyzed using the Shapiro–Wilk test to assess normal distribution and the Levene test to evaluate the homogeneity of variances. Depending on the results of these tests, different statistical methods were applied: Tukey’s honest significance test (HSD) for normally distributed and homogeneous data, T3 Dunnett test for normally distributed but non-homogeneous data, and Kruskal–Wallis test for non-normally distributed data. IBM SPSS Statistics software v.22 (IBM Co., New York, NY, USA) was utilized for these analyses. RATA data were utilized to construct a CATA-type binary matrix, and the results were analyzed based on attribute citations. For attributes cited more than 20%, CATA results were analyzed using Cochran’s Q test, Bonferroni pairwise multiple comparisons, Correspondence Analysis (CA), and Penalty Analysis (PA) with linking data via XLSTAT 2023.2.0 software (Addinsoft, Paris, France). This software was also employed to conduct Pearson correlation tests among the physicochemical and texture characteristics of each sample in this study. Statistical significance for all analyses was determined using a 5% level of significance (*p* ≤ 0.05).

## 3. Results and Discussion

### 3.1. Physicochemical Characteristics

Banana pH values on the initial day started at 4.55–4.77 and at the final fermentation day were found between 4.41 and 4.97, with no statistical differences between both results (Table 1). Apple pH values began around 3.45 and finished between 2.72 and 3.26, with lower values (*p* ≤ 0.05) in the LAB-inoculated batches in comparison with the initial fermentation day (Table 2). The pH values of both fruits confirmed that they were correctly ripened according to the literature [30,31]. The modification of salt provoked no differences in the pH values between the LAB-inoculated batches on bananas, though these batches were slightly different on the fermented apple, with lower values (*p* ≤ 0.05) in the salt-reduced batch, conditions that could allow the LAB to be more effective [32].

Fermentation did affect the a_w_ of the products, as it decreased (*p* ≤ 0.05) in all the analyzed batches for both fruits on the final day of fermentation. Bananas showed starting values of 0.982–0.994 and final values between 0.961 and 0.974 (Table 1). Apple displayed initial values around 0.990 that decreased to 0.975–0.978 (Table 2). All the final banana a_w_ decreased more (*p* ≤ 0.05) in the 2% salt concentrations batches, which is related to the NaCl capacity to decrease this parameter. However, in the apple, no differences were found between the batches with different salt concentrations, which might be linked to the activity of LAB to reduce a_w_ due to the production of metabolites, such as organic acids [33].

The °Brix of banana was 20.10 on the initial day and increased to 21.00–21.50 at the final fermentation. Apple started at 13.33–13.78 and decreased to 11.78–13.10 during fermentation. The initial total soluble solid concentration of both fruits coincides with the ones found in the bibliography [34,35]. A decrease in °Brix was expected due to the utilization of simple sugars during fermentation [36]. However, in the case of bananas, no °Brix reduction was found, which might be related to the non-reduction in pH and the two days of fermentation in this fruit.

### 3.2. Microbial Results

The counts of lactic acid bacteria were detected from around 6.50 log CFU/g to 5.58–7.46 log CFU/g in the case of banana during the two days of fermentation (Table 3). Almost every analyzed batch showed statistical differences (*p* ≤ 0.05) between the initial and the final sampling day, decreasing in the case of the batches without LAB inoculation and increasing in the case of the *L. paracasei*-inoculated batches. Inoculated LAB increased during ripened banana fermentation, as already validated by other authors [37], so it could be understood that wild bacteria found in bananas were not adapted to the fermentation conditions. However, inoculated bananas did adapt to this fruit as can be observed in the higher LAB concentrations achieved at the final sampling day in the LAB-inoculated batches, in comparison to the LAB-uninoculated batches for every salt concentration evaluated. Moreover, *L. paracasei* inoculation achieved yeast growth control in the *E. coli* non-inoculated batches.

In the case of apple, the counts were detected on the initial day between 5.20 and 5.93 log CFU/g, and, on the final day, reached values between 6.51 and 7.44 log CFU/g (Table 4). Most of the batches displayed statistical differences (*p* ≤ 0.05) between both sampling days, although in this fruit, all of them increased during the seven days of fermentation. Lactic fermented apples showed increases in viable bacteria along the fermentation time in other studies, as it seems that this fruit is an ideal raw material for LAB growth [38,39]. Compared to bananas, the 7-day fermentation in this matrix allows the microorganisms to adapt even though their lag phase is longer and begin the exponential phase before the end of the 7 days. The inoculation of *L. paracasei* achieved higher LAB counts on the final sampling day for each of the evaluated salt concentrations.

Both fruits reached values over 7.00 log CFU/g only in the *L. paracasei*-inoculated batches, which could be justified due to the adaptation of the strain employed for the fermentation process in contrast with the wild LAB present in the product. It is also remarkable that on the final banana fermentation day, every batch showed lower LAB counts (*p* ≤ 0.05) in the 2% salt batches than in the salt-eliminated ones. This fact is shared by some yeast counts in the final fermentation day of bananas too. This is in line with the research of other authors who show how microorganisms can grow better with lower salt concentrations [33].

The evaluation of yeast development in fermented products is important because this microbial group generally produces alcoholic fermentation and might induce off-flavors in the final product [22,40]. Yeast counts were detected from around 4.60 log CFU/g to 5.48–6.36 log CFU/g in the case of bananas during the two fermentation days (Table 3). All the analyzed batches displayed statistical differences (*p* ≤ 0.05) between the initial and the final sampling day. In the case of apples, the counts were detected on the initial day around 5.60 log CFU/g, and on the seventh day, reached values between 5.85 and 6.41 log CFU/g (Table 4). Most of the batches displayed statistical differences (*p* ≤ 0.05) between both sampling days, though all of them increased during the seven days of fermentation. Bananas and apples are fruits that are usually yeast-fermented to produce wine or cider, so their yeast population is known to be able to develop in these raw materials [41,42]. However, the apple inoculation of *L. paracasei* provided a control tool for the growth of yeasts in the LAB-inoculated batches, although statistical differences were not found in all cases. Furthermore, the yeast control that takes place in *L. paracasei*-inoculated batches in both fruits (Table 3 and Table 4) may contribute to a reduction in undesired sensory characteristics. Biocontrol might be related to the competition for nutrients or the inhibition by LAB-produced metabolites, as it has been reported that the yeasts might exhaust nutrients in the batches in which LAB was not inoculated [43,44].

*E. coli* O157:H7, the pathogen microorganism used to assess the safety of the process, was found only in the batches in which it was inoculated (Table 3 and Table 4). In both fruits, the initial microbial load was around 2.10 log CFU/g, and in both cases, the highest results (*p* ≤ 0.05) were found on the final day of fermentation in the salt-reduced batches, with an increase over 1.50 log CFU/g. In the case of bananas, growth in *E. coli* was observed (*p* ≤ 0.05) in every analyzed batch except for the one inoculated with *L. paracasei* and 2% salt (Table 3). The pH and a_w_ values of this batch did not present differences with the 2% salt uninoculated batch (Table 1), so the inclusion of *L. paracasei* is the remaining differential parameter that explains the protective effect observed. In the case of apple, the pathogenic bacterium was only able to develop in the *L. paracasei* uninoculated batches, proving how the strain employed might be an appropriate strategy to avoid *E. coli* bacterial development, even in the salt-reduced batches (Table 4). Moreover, *E. coli* reductions were achieved in the EC+LAB 1% batch on the final fermentation day in comparison with the *L. paracasei* uninoculated analog, revealing the ability of these LAB to control the pathogen microorganism under reduced salt conditions. Indeed, the lowest pH was achieved in the salt-reduced *L. paracasei*-inoculated batch, demonstrating the effectiveness of the strain employed even under salt-reduced conditions. According to other authors, the temperature of fermentation in the present study is optimal to promote the growth of *E. coli*, and the lowest salt concentration is a key factor in promoting fast development [45]. However, it is remarkable how higher *E. coli* counts (*p* ≤ 0.05) were observed in the apple LAB-uninoculated batch at 1% salt in contrast to its analog at 2%, but these differences are not displayed in the LAB-inoculated batches. In bananas, *L. paracasei* also presents a protective effect, but it is only observed in combination with the 2% salt concentration. It is essential to note that apple fermentation time was 7 days, and bananas were fermented for 2 days, so the effectiveness of the *L. paracasei* biocontrol might even display higher *E. coli* reduction rates in bananas if longer fermentation is performed, as occurred for apples. The employment of *L. paracasei* as a protective agent against *E. coli* has already been evaluated by other authors, and future perspectives of the present study might evaluate if the protection is performed due to a competition of nutrients or the production of antimicrobial metabolites [46,47]. However, this study was performed under specific conditions, and a physicochemical, sensory, and safety study must be performed for each case if any parameter is modified. Future research should explore the mechanisms behind the protective effects of *L. paracasei* and investigate its applicability to a wider range of fermented fruit products.

### 3.3. Sensory Evaluation

The Check-All-That-Apply analysis performed on fermented bananas showed that, among the 23 terms evaluated, the most frequently used by consumers were as follows: fermented odor/flavor; moisty texture; stale fruit odor; oxidation appearance; acid taste; alcoholic flavor, etc. These terms can be considered the most appropriate in the description of the samples by the participants. Lower frequencies of bitter, salty, and off-flavor were identified in the salt-reduced batch in comparison with the 2% salt batch (Table 5). However, the uninoculated batches displayed a higher identification of cooked fruit flavor and sweet taste than the one inoculated with *L. paracasei*, which was identified as off-flavor with more frequency than the former batch. The lactic fermentation of some vegetables may develop compounds identified as off-flavor, as evaluated in watermelon juice, soymilk, or walnuts [29]. To the best of our knowledge, no studies have been performed on bananas.

When measuring the intensity of these attributes using the RATA method, in general, the most frequent attributes are also the most intense. No attribute presented an average intensity greater than 3, which indicates medium intensity. The hedonic test also proved that the salt elimination strategy can improve (*p* ≤ 0.05) the quality of bananas if *L. paracasei* is not inoculated (Table 6). The lowest liking was found in the 2% batch, and the highest punctuation was achieved by 0%. The 0% batch is the one most microbiologically compromised, and extreme hygienic conditions must be applied to avoid possible *E. coli* contamination that could grow during the fermentation process, as can be observed in Table 3.

**Figure 2:**
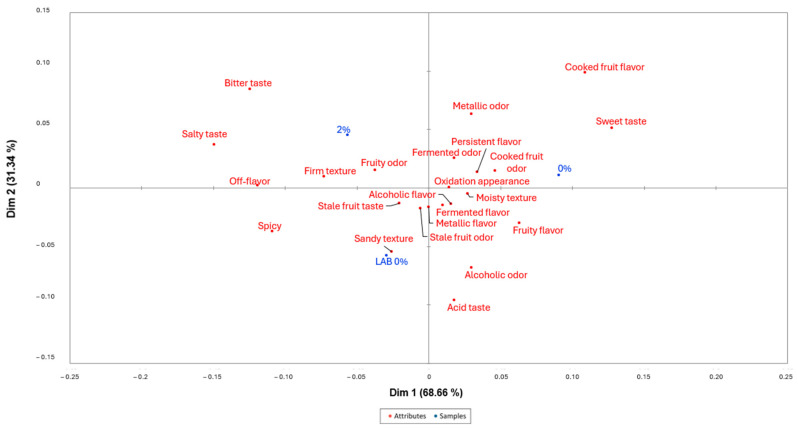
Correspondence Analysis of the evaluated batches of fermented bananas and the selected attributes. Batch 2% (2% salt), 0% (0% salt), LAB 0% (0% salt inoculated with *Lacticaseibacillus paracasei*). Batches are displayed in blue color and attributes in red color.

Figure 2 shows a projective map for the fermented banana correspondence analysis (CA). The first dimension explained 68.66% of the total variance and the second dimension was 31.34%. All the samples were in different quadrants, showing a different sensory map. Batch 0% was placed with positive values of the first and second dimensions, being mainly described as cooked fruit flavor, and sweet taste. Batch 2% was located at negative values in the first dimension and positive values in the second, being mainly characterized by a salty and bitter taste, firm texture, and fruity odor. Batch LAB 0% was placed with negative values of the first and second dimensions, mainly characterized by a spicy, sandy texture and stale fruit taste. Lactic acid fermentation might produce aldehydes or other volatile molecules identified as off-flavors in some fruits, which might explain why the inoculation of LAB in bananas reduces the quality of the final product [48].

**Figure 3:**
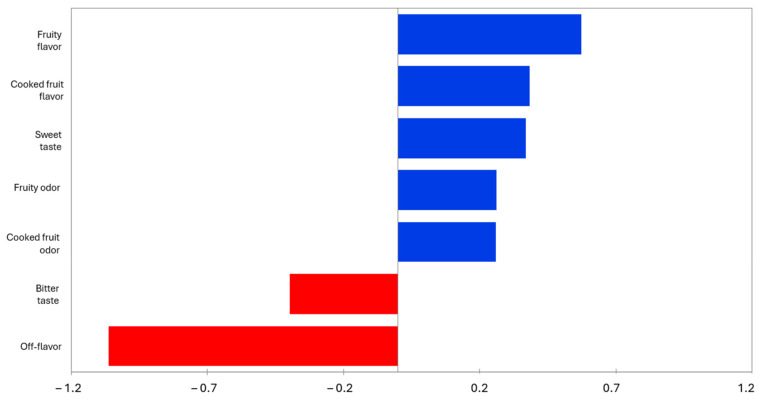
Penalty Analysis of fermented banana. Blue filling is employed to display the attributes that positively affect the average, meanwhile red filling is employed to show the attributes that negatively affect the average.

Figure 3 shows the mean drops in liking as a function of the proportion of consumers that checked an attribute differently than for the fermented banana. As shown, the penalty analysis allowed us to identify off-flavor and bitter taste, negatively affecting the acceptability. These attributes are related to batch 2% according to Figure 2. On the other hand, fruity flavor, cooked fruit flavor, sweet taste, fruity odor, and cooked fruit odor are attributes that positively affect the acceptability of the product. Most of these attributes are found closer to batch 0% in Figure 2, justifying the highest acceptability of this batch (Table 6). The addition of salt might select wild LAB that produce lactic acid fermentation in bananas, reducing the quality of the final product as a result of the production of non-desirable volatile compounds [48].

Fermented apple CATA analysis showed lower (*p* ≤ 0.05) sweetness and higher (*p* ≤ 0.05) alcoholic odor and flavor as well as salty taste and off-flavor in the uninoculated batch with 2% salt than in the salt-reduced batches (Table 7). Moreover, the off-flavor might be specified in the attributes that displayed higher citation values (*p* ≤ 0.05), stale fruit odor and flavor, fermented odor and flavor, and spicy, in comparison to the salt-reduced apple inoculated with *L. paracasei*. A controlled fermentation can achieve a quality fermented apple with the correct aroma development [49].

When measuring the intensity of these attributes using the RATA method, generally, the most frequent attributes were also the most intense. No attribute presented an average intensity greater than 3, which indicates medium intensity. The hedonic test showed the 2% salt batch as the one with the worst (*p* ≤ 0.05) acceptability (Table 8). In apple, higher quality was achieved in the LAB-inoculated batch, which furthermore controlled *E. coli,* as shown in Table 4. The employment of *L. paracasei* in fruit fermentation has, thus, been confirmed as a protective strategy that can improve the fruit’s quality. This may be promising for other fruits, although a specific evaluation must be carried out to assess whether the characteristics of each fruit and fermentation process allow for the effectiveness of the inoculated LAB.

**Figure 4:**
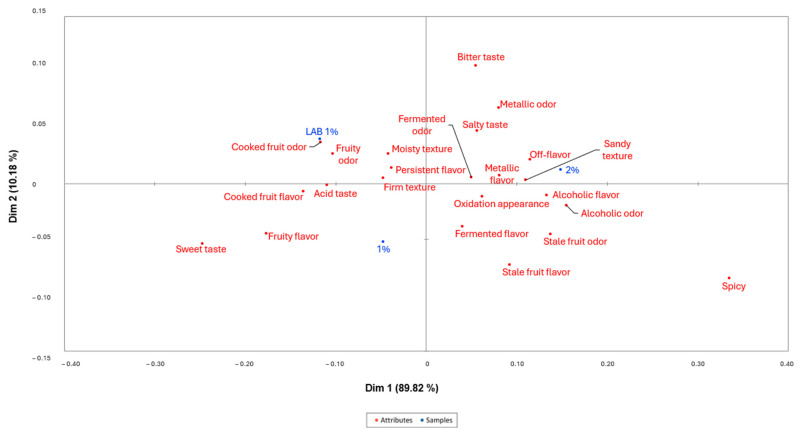
Correspondence Analysis of the evaluated batches of fermented apple and the selected attributes. Batch 2% (2% salt), 1% (1% salt), LAB 1% (1% salt inoculated with *Lacticaseibacillus paracasei*). Batches are displayed in blue color and attributes in red color.

Figure 4 displays a projective map for the fermented apple Correspondence Analysis (CA). The first dimension explained 89.82% of the total variance and the second dimension was 10.18%. All the samples were identified in different quadrants, showing a different sensory map. According to Figure 4, batch 2% was placed with positive values for both dimensions, showing a stronger relationship with attributes, such as off-flavor, stale fruit flavor and odor, sandy texture, fermented flavor and odor, metallic flavor and odor, oxidation appearance, bitter and salty taste. Batch LAB 1% was located at negative values of the first dimension and positive values of the second one. Batch 1% was placed with negative values for both dimensions. Batches 1% and LAB 1% are closer to sweet taste, fruity flavor, and odor. In apple, the utilization of *L. paracasei* produced a change in the final product that enhances its quality, though the selection of wild LAB in batch 2% may have generated non-desirable compounds [48].

**Figure 5:**
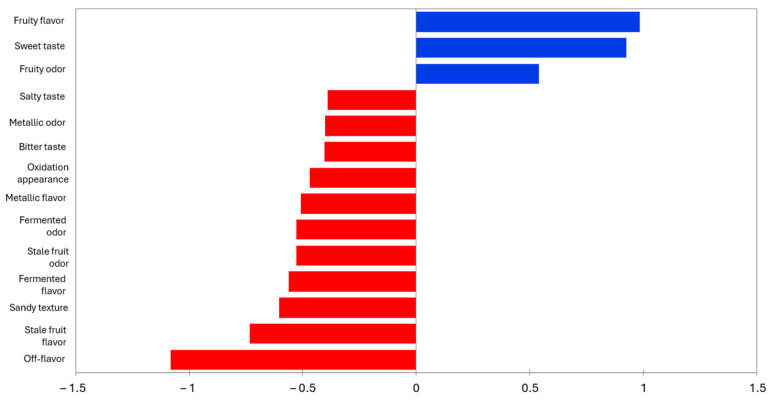
Penalty Analysis of fermented apples. Blue filling is employed to display the attributes that positively affect the average, meanwhile red filling is employed to show the attributes that negatively affect the average.

Figure 5 shows the mean drops in liking as a function of the proportion of consumers that checked an attribute differently than for the fermented apple. As shown, the penalty analysis allowed us to identify fruity flavor, sweet taste, and fruity odor as attributes that positively affect the liking of the product. These attributes are shared by batches 1% and LAB 1%, as shown in Figure 4, though higher values were identified in the RATA analysis for batch LAB 1% (Table 8). On the other hand, salty taste, metallic odor, bitter taste, oxidation appearance, metallic flavor, fermented odor, stale fruit odor, fermented flavor, sandy texture, stale fruit flavor, and off-flavor are the attributes that PA identified as acceptability reducers. Batch 2% is identified in Figure 4 with all these attributes and could reduce its liking up to 1 point on a 7-point scale.

The fermentation of fruits is not only a strategy to increase the lifetime value of a product but also a way to develop different sensory characteristics or introduce health benefits to our diet through accessible fruits or commodity products [50]. Fruit fermentation can drive beneficial agents such as antioxidants, postbiotics, or probiotics into daily diets [37,49]. The present study justified that a salt reduction in fermented bananas and apples improves the liking of both fruits, as the sodium intake is reduced to improve consumer health. In apples, the employment of selected LAB as *L. paracasei* is necessary to develop a high-quality final product.

## 4. Conclusions

Salt reduction and fermentation with *L. paracasei* are a promising strategy for the sensory and microbiological quality of fermented fruits. The intrinsic characteristics of the fruits must be considered to ensure the method’s efficiency since different impacts are observed in apples and bananas. The elimination of salt improved sensory qualities but compromised microbiological safety, allowing *E. coli* growth in fermented bananas. In contrast, for apples, *L. paracasei* inoculation effectively controlled *E. coli* growth, even in salt-reduced conditions (1% salt). This treatment also enhanced sensory qualities, reducing off-flavors and improving overall acceptability compared to the 2% salt batch. The sensory evaluation highlighted the complex interplay between salt reduction, LAB inoculation, and consumer perception. While salt reduction generally improved acceptability, the impact of *L. paracasei* on sensory attributes varied between fruits. Therefore, the LAB can be proposed for use in the fermentation process of apples, being an effective, cheap, and feasible tool to enhance the quality and safety of this process. Future steps will consist of the fermentation of different fruits and vegetables to evaluate their possibility to reduce the sodium content.

## Figures and Tables

**Table 1:** Physicochemical characteristics of bananas at the initial and final day of fermentation.

	Batch ^•^	pH	a_w_	°Brix
Day 0	0%	4.77 ± 0.28	0.994 ± 0.003 *°	20.10 ± 0.95
	LAB 0%	4.77 ± 0.28	0.994 ± 0.003 *°	20.10 ± 0.95
	2%	4.55 ± 0.52	0.982 ± 0.007 *°	20.10 ± 0.65 *
	LAB 2%	4.55 ± 0.52	0.982 ± 0.007 *°	20.10 ± 0.65
Day 2	0%	4.97 ± 0.14 ^2^°	0.970 ± 0.008 ^12^*°	21.47 ± 1.13
	LAB 0%	4.45 ± 0.75 ^12^	0.974 ± 0.007 ^2^*°	21.27 ± 0.85
	2%	4.42 ± 0.21 ^1^°	0.961 ± 0.006 ^1^*°	21.50 ± 1.23 *
	LAB 2%	4.41 ± 0.12 ^1^	0.966 ± 0.005 ^12^*°	21.00 ± 0.87

Batch 0% (0% salt), LAB 0% (0% salt inoculated with *Lacticaseibacillus paracasei*), 2% (2% salt), LAB 2% (2% salt inoculated with *L. paracasei*). Significant differences (*p* ≤ 0.05) were analyzed among the different days from the same sampling day and analysis (^1,2^), between the initial and final day for each batch and analysis (*), between the *L. paracasei* inoculated and uninoculated batches within the same day, analysis, and salt concentration (^•^), and between the salt concentrations for the same batch, day and analysis (°).

**Table 2:** Physicochemical characteristics of apples at the initial and final day of fermentation.

	Batch	pH	a_w_	°Brix
Day 0	1%	3.48 ± 0.22	0.992 ± 0.005 *	13.33 ± 1.47
	LAB 1%	3.48 ± 0.22 *	0.992 ± 0.005 *	13.33 ± 1.47
	2%	3.41 ± 0.30	0.989 ± 0.003 *	13.78 ± 1.92
	LAB 2%	3.41 ± 0.30 *	0.989 ± 0.003 *	13.78 ± 1.92
Day 7	1%	3.26 ± 0.28 ^2•^	0.978 ± 0.008 *	13.10 ± 4.20
	LAB 1%	2.72 ± 0.04 ^1^*^•^°	0.975 ± 0.005 *	11.78 ± 5.29
	2%	3.13 ± 0.33 ^1,2^	0.975 ± 0.004 *	12.02 ± 4.63
	LAB 2%	2.89 ± 0.05 ^2^*°	0.975 ± 0.004 *	12.32 ± 5.27

Batch 1% (1% salt), LAB 1% (1% salt inoculated with *Lacticaseibacillus paracasei*), 2% (2% salt), LAB 2% (2% salt inoculated with *L. paracasei*). Significant differences (*p* ≤ 0.05) were analyzed among the different days from the same sampling day and analysis (^1,2^), between the initial and final day for each batch and analysis (*), between the *L. paracasei* inoculated and uninoculated batches within the same day, analysis, and salt concentration (^•^), and between the salt concentrations for the same batch, day and analysis (°).

**Table 3:** Microbial banana counts (log CFU/g) of *Escherichia coli* O157:H7, lactic acid bacteria, and yeasts for the initial and final day of fermentation.

	Batch	*Escherichia coli*	Lactic Acid Bacteria	Yeasts
Day 0	0%	<1	6.44 ± 0.38 *	4.73 ± 0.27 *
	LAB 0%	<1	6.79 ± 0.42 *	4.53 ± 0.25 *
	EC 0%	2.07 ± 0.48 *	6.44 ± 0.38 *	4.72 ± 0.27 *
	EC+LAB 0%	2.07 ± 0.48*	6.79 ± 0.42 *	4.53 ± 0.25 *
	2%	<1	6.44 ± 0.38 *	4.73 ± 0.27 *
	LAB 2%	<1	6.79 ± 0.42	4.53 ± 0.25 *
	EC 2%	2.07 ± 0.48 *	6.44 ± 0.38 *	4.73 ± 0.27 *
	EC+LAB 2%	2.07 ± 0.48	6.79 ± 0.42 *	4.53 ± 0.25 *
Day2	0%	<1	5.95 ± 0.13 °*^1,2•^	6.46 ± 0.01 °*^3•^
	LAB 0%	<1	7.46 ± 0.10 °*^3•^	6.09 ± 0.20 °*^1,2,3•^
	EC 0%	3.68 ± 0.52 *^2^	5.96 ± 0.54 °*^1,2,3•^	6.19 ± 0.20 *^1,2,3^
	EC+LAB 0%	3.26 ± 0.42 *^1,2^	7.42 ± 0.72 °*^2,3•^	6.12 ± 0.39 *^1,2,3^
	2%	<1	5.63 ± 0.57 °*^1•^	6.36 ± 0.11 °*^2•^
	LAB 2%	<1	6.83 ± 0.15 °^1,2,3•^	5.48 ± 0.45 °*^1•^
	EC 2%	2.98 ± 0.73 *^1,2^	5.58 ± 0.81 °*^1•^	6.09 ± 0.28 *^1,2,3^
	EC+LAB 2%	2.39 ± 0.92 ^1^	7.25 ± 0.12 °*^2,3•^	5.83 ± 0.33 *^1,2^

Batch 0% (0% salt), LAB 0% (0% salt inoculated with *Lacticaseibacillus paracasei*), EC 0% (0% salt inoculated with *E. coli*), EC+LAB 0% (0% salt inoculated with *E. coli* and *L. paracasei*), 2% (2% salt), LAB 2% (2% salt inoculated with *L. paracasei*), EC 2% (2% salt inoculated with *E. coli*), EC+LAB 2% (2% salt inoculated with *E. coli* and *L. paracasei*). Significant differences (*p* ≤ 0.05) were analyzed among the different days from the same sampling day and microbial group (^1−3^), between the initial and final day for each batch and microbial group (*), between the *L. paracasei* inoculated and uninoculated batches within the same day, analysis, and salt concentration (^•^), and between the salt concentrations for the same batch, day and microbial group (°).

**Table 4:** Microbial apple counts (log CFU/g) of *Escherichia coli* O157:H7, lactic acid bacteria, and yeasts for the initial and final day of fermentation.

	Batch	*Escherichia coli*	Lactic Acid Bacteria	Yeasts
Day 0	1%	<1	5.20 ± 0.19 *^1•^	5.75 ± 0.14 *^•^
	LAB 1%	<1	5.93 ± 0.34 *^2•^	5.50 ± 0.14 *^•^
	EC 1%	2.17 ± 0.48 *	5.20 ± 0.19 *^1•^	5.75 ± 0.14 *^•^
	EC+LAB 1%	2.17 ± 0.48	5.93 ± 0.34 *^2•^	5.50 ± 0.14 *^•^
	2%	<1	5.20 ± 0.19 *^1•^	5.75 ± 0.14 *^•^
	LAB 2%	<1	5.93 ± 0.34 ^2•^	5.50 ± 0.14 *^•^
	EC 2%	2.17 ± 0.48 *	5.20 ± 0.19 *^1•^	5.75 ± 0.14 *^•^
	EC+LAB 2%	2.17 ± 0.48	5.93 ± 0.34 *^2•^	5.50 ± 0.14^•^
Day 7	1%	<1	6.81 ± 0.55 *^1,2•^	6.34 ± 0.18 *
	LAB 1%	<1	7.43 ± 0.35 *^2•^	6.17 ± 0.16 *
	EC 1%	3.74 ± 0.67 °*^2•^	6.72 ± 0.50 *^1,2•^	6.32 ± 0.25 *
	EC+LAB 1%	1.49 ± 0.27 ^1,2•^	7.44 ± 0.42 *^2•^	6.19 ± 0.30 *
	2%	<1	6.76 ± 0.54 *^1,2•^	6.32 ± 0.19 *
	LAB 2%	<1	7.42 ± 0.60 ^2•^	6.13 ± 0.34 *
	EC 2%	2.69 ± 0.23 °*^1^	6.51 ± 0.31 *^1•^	6.41 ± 0.47 *^•^
	EC+LAB 2%	1.55 ± 1.28 ^1^	7.07 ± 0.45 *^1,2•^	5.85 ± 0.47 ^•^

Batch 1% (1% salt), LAB 1% (1% salt inoculated with *Lacticaseibacillus paracasei*), EC 1% (1% salt inoculated with *E. coli*), EC+LAB 1% (1% salt inoculated with *E. coli* and *L. paracasei*), 2% (2% salt), LAB 2% (2% salt inoculated with *L. paracasei*), EC 2% (2% salt inoculated with *E. coli*), EC+LAB 2% (2% salt inoculated with *E. coli* and *L. paracasei*). Significant differences (*p* ≤ 0.05) were analyzed among the different days from the same sampling day and microbial group (^1,2^), between the initial and final day for each batch and microbial group (*), between the *L. paracasei* inoculated and uninoculated batches within the same day, analysis, and salt concentration (^•^), and between the salt concentrations for the same batch, day and microbial group (°).

**Table 5:** Frequencies (%) of citation of attributes from the Check-All-That-Apply for fermented bananas.

Attributes	2%	0%	LAB 0%
Oxidation appearance	88.24	85.29	85.29
Stale fruit odor	92.65	86.76	92.65
Cooked fruit odor	54.41	55.88	51.47
Fruity odor	67.65	57.35	61.76
Metallic odor	61.76	58.82	51.47
Fermented odor	98.53	94.12	89.71
Alcoholic odor	69.12	73.53	79.41
Stale fruit flavor	89.71	80.88	88.24
Cooked fruit flavor	60.29 ^1,2^	66.18 ^2^	47.06 ^1^
Fruity flavor	66.18	73.53	70.59
Metallic flavor	57.35	54.41	57.35
Fermented flavor	94.12	91.18	94.12
Alcoholic flavor	76.47	75.00	76.47
Firm texture	33.82	26.47	30.88
Sandy texture	36.76	33.82	39.71
Moisty texture	98.53	98.53	97.06
Persistent flavor	79.41	79.41	75.00
Spicy	33.82	25.00	33.82
Acid taste	73.53	77.94	89.71
Sweet taste	54.41 ^1,2^	64.71 ^2^	48.53 ^1^
Bitter taste	60.29 ^2^	39.71 ^1^	45.59 ^1,2^
Salty taste	80.88 ^2^	51.47 ^1^	67.65 ^1,2^
Off-flavor	83.82 ^2^	58.82 ^1^	76.47 ^2^

Batch 2% (2% salt), 0% (0% salt), LAB 0% (0% salt inoculated with *Lacticaseibacillus paracasei*). Data are expressed as mean citation frequency. Statistical differences (*p* ≤ 0.05) between batches are displayed with numbers as superscripts (^1,2^).

**Table 6:** Rate-All-That-Apply sensory results for fermented bananas over a five-point scale.

Attributes	2%	0%	LAB 0%
Oxidation appearance	1.68 ± 0.98	1.62 ± 1.01	1.46 ± 0.92
Stale fruit odor	1.87 ± 0.91	1.69 ± 1.03	1.68 ± 0.87
Cooked fruit odor	0.74 ± 0.78	0.72 ± 0.77	0.69 ± 0.78
Fruity odor	0.99 ± 0.87	0.75 ± 0.76	0.99 ± 0.97
Metallic odor	0.93 ± 0.94	0.97 ± 1.01	0.84 ± 0.99
Fermented odor	2.25 ± 0.76 ^2^	1.97 ± 0.83 ^1,2^	1.84 ± 0.94 ^1^
Alcoholic odor	1.25 ± 1.04	1.24 ± 0.95	1.21 ± 0.84
Stale fruit flavor	1.79 ± 1.00	1.60 ± 1.08	1.71 ± 0.95
Cooked fruit flavor	0.84 ± 0.84	1.01 ± 0.94	0.65 ± 0.79
Fruity flavor	0.90 ± 0.78	1.16 ± 0.91	0.96 ± 0.80
Metallic flavor	0.96 ± 1.00	0.82 ± 0.91	1.01 ± 1.04
Fermented flavor	2.15 ± 0.85	1.91 ± 0.94	2.04 ± 0.87
Alcoholic flavor	1.28 ± 0.91	1.26 ± 0.94	1.34 ± 0.97
Firm texture	0.43 ± 0.65	0.34 ± 0.61	0.41 ± 0.72
Sandy texture	0.53 ± 0.82	0.51 ± 0.82	0.60 ± 0.90
Moisty texture	2.40 ± 0.72	2.38 ± 0.71	2.29 ± 0.77
Persistent flavor	1.31 ± 0.97	1.21 ± 0.89	1.29 ± 1.01
Spicy	0.56 ± 0.92	0.38 ± 0.75	0.59 ± 0.95
Acid taste	1.43 ± 1.06	1.35 ± 0.97	1.76 ± 0.95
Sweet taste	0.68 ± 0.74 ^1,2^	1.06 ± 0.94 ^2^	0.66 ± 0.78 ^1^
Bitter taste	1.06 ± 1.06 ^2^	0.63 ± 0.91 ^1^	0.74 ± 0.99 ^1,2^
Salty taste	1.66 ± 1.09 ^2^	0.75 ± 0.87 ^1^	1.06 ± 0.91 ^1^
Off-flavor	1.75 ± 1.08 ^2^	1.15 ± 1.18 ^1^	1.44 ± 1.10 ^1,2^
Acceptability	2.12 ± 1.19 ^1^	2.71 ± 1.22 ^2^	2.40 ± 1.31 ^1,2^

Batch 2% (2% salt), 0% (0% salt), LAB 0% (0% salt inoculated with *Lacticaseibacillus paracasei*). Data are expressed as mean ± standard deviation. Statistical differences (*p* ≤ 0.05) between batches are displayed with numbers as superscripts (^1,2^).

**Table 7:** Frequencies (%) of citation of attributes from the Check-All-That-Apply for fermented apple.

Attributes	2%	1%	LAB 1%
Oxidation appearance	49.28	42.03	37.68
Stale fruit odor	72.46 ^2^	57.97 ^1,2^	44.93 ^1^
Cooked fruit odor	57.97	62.32	69.57
Fruity odor	65.22	69.57	75.36
Metallic odor	46.38	33.33	36.23
Fermented odor	92.75 ^2^	78.26 ^1,2^	73.91 ^1^
Alcoholic odor	85.51 ^2^	63.77 ^1^	52.17 ^1^
Stale fruit flavor	71.01 ^2^	63.77 ^1,2^	47.83 ^1^
Cooked fruit flavor	56.52	68.12	69.57
Fruity flavor	55.07 ^1^	76.81 ^2^	73.91 ^1,2^
Metallic flavor	50.72	40.58	37.68
Fermented flavor	95.65 ^2^	88.41 ^1,2^	75.36 ^1^
Alcoholic flavor	78.26 ^2^	59.42 ^1^	50.72 ^1^
Firm texture	89.86	89.86	89.86
Sandy texture	37.68	28.99	26.09
Moisty texture	91.30	86.96	91.30
Persistent flavor	88.41	85.51	86.96
Spicy	20.29 ^2^	13.04 ^1,2^	7.25 ^1^
Acid taste	65.22	73.91	75.36
Sweet taste	42.03 ^1^	69.57 ^2^	68.12 ^2^
Bitter taste	47.83	33.33	40.58
Salty taste	84.06 ^2^	65.22 ^1^	68.12 ^1^
Off-flavor	66.67 ^2^	49.28 ^1^	46.38 ^1^

Batch 2% (2% salt), 1% (1% salt), LAB 1% (1% salt inoculated with *Lacticaseibacillus paracasei*). Data are expressed as mean citation frequency. Statistical differences (*p* ≤ 0.05) between batches are displayed with numbers as superscripts (^1,2^).

**Table 8:** Rate-All-That-Apply sensory results for fermented apples over a five-point scale.

Attributes	2%	1%	LAB 1%
Oxidation appearance	0.83 ± 0.98	0.64 ± 0.86	0.55 ± 0.74
Stale fruit odor	1.35 ± 1.07 ^2^	0.88 ± 0.92 ^1^	0.62 ± 0.82 ^1^
Cooked fruit odor	0.80 ± 0.81	0.87 ± 0.82	1.17 ± 0.91
Fruity odor	0.96 ± 0.91 ^1^	1.06 ± 0.91 ^1,2^	1.42 ± 1.03 ^2^
Metallic odor	0.75 ± 0.93	0.49 ± 0.80	0.50 ± 0.70
Fermented odor	1.97 ± 0.86 ^2^	1.30 ± 0.93 ^1^	1.12 ± 0.91 ^1^
Alcoholic odor	1.88 ± 1.05 ^2^	1.09 ± 1.04 ^1^	0.79 ± 0.90 ^1^
Stale fruit flavor	1.22 ± 1.00 ^2^	0.97 ± 0.92 ^1,2^	0.65 ± 0.83 ^1^
Cooked fruit flavor	0.75 ± 0.79	0.94 ± 0.82	1.12 ± 0.94
Fruity flavor	0.83 ± 0.89 ^1^	1.25 ± 0.91 ^2^	1.42 ± 1.05 ^2^
Metallic flavor	0.81 ± 0.96	0.62 ± 0.93	0.65 ± 0.95
Fermented flavor	1.93 ± 0.81 ^2^	1.52 ± 0.85 ^1^	1.18 ± 0.93 ^1^
Alcoholic flavor	1.70 ± 1.14 ^2^	1.01 ± 1.05 ^1^	0.71 ± 0.90 ^1^
Firm texture	1.80 ± 0.87	1.74 ± 0.87	1.80 ± 0.93
Sandy texture	0.48 ± 0.68	0.38 ± 0.64	0.42 ± 0.79
Moisty texture	1.71 ± 0.84	1.55 ± 0.93	1.55 ± 0.87
Persistent flavor	1.52 ± 0.85	1.36 ± 0.84	1.35 ± 0.76
Spicy	0.30 ± 0.69	0.20 ± 0.58	0.11 ± 0.43
Acid taste	1.16 ± 1.02	1.09 ± 0.87	1.15 ± 0.87
Sweet taste	0.55 ± 0.76 ^1^	0.97 ± 0.80 ^2^	1.05 ± 0.86 ^2^
Bitter taste	0.77 ± 0.97	0.42 ± 0.67	0.62 ± 0.88
Salty taste	1.59 ± 1.00 ^2^	1.01 ± 0.95 ^1^	1.03 ± 0.95 ^1^
Off-flavor	1.38 ± 1.15 ^2^	0.77 ± 0.96 ^1^	0.62 ± 0.84 ^1^
Acceptability	2.58 ± 1.23 ^1^	3.26 ± 1.17 ^2^	3.55 ± 1.12 ^2^

Batch 2% (2% salt), 1% (1% salt), LAB 1% (1% salt inoculated with *Lacticaseibacillus paracasei*). Data are expressed as mean ± standard deviation. Statistical differences (*p* ≤ 0.05) between batches are displayed with numbers as superscripts (^1,2^).

## Data Availability

The original contributions presented in the study are included in the article; further inquiries can be directed to the corresponding author.
